# Rapid Expansion of the Eastern China Lineage (Haplotype P) of *Phragmites australis* in Caohai Lake, Guizhou, China

**DOI:** 10.1002/ece3.73711

**Published:** 2026-05-21

**Authors:** Wangjun Li, Lele Lin, Yuhui Wang, Wenlong Sun, Weihua Guo, Xiaolong Bai, Lele Liu

**Affiliations:** ^1^ Guizhou Key Laboratory of Plateau Wetland Conservation and Restoration Guizhou University of Engineering Science Bijie China; ^2^ Ecology and Nature Conservation Institute Chinese Academy of Forestry Beijing China; ^3^ National Glycoengineering Research Center Shandong University Qingdao China; ^4^ School of Life Sciences Shandong University Qingdao China

**Keywords:** cryptic invasions, *Phragmites australis*, plant invasion, plateau lake

## Abstract

*Phragmites australis* is a well‐known invasive grass in North America, but its rapid expansion into new habitats within its native range is less documented. Here, we report a rapid expansion event of *P. australis* over the past decade in Caohai Lake, a plateau lake in China. Remote sensing and field surveys revealed that the reed population now covers 454.09 ha, constituting 3.8% of the protected area (approximately half of the total lake shoreline), with the most significant expansion occurring between 2018 and 2020. Ecological niche modeling with the previous records in China indicated a low habitat suitability (index = 0.354) for *P. australis* in this region. Despite this, our genetic analysis identified that all sampled individuals belong to haplotype P, an introduced octoploid lineage from eastern China. This finding excludes a natural range expansion from native populations in northern or southwestern China. The expansion is likely driven by anthropogenic habitat modification (e.g., “returning farmland to wetland”) and the introduction of this competitive genotype via constructed wetlands. Our study underscores the risk of “cryptic invasions” by introduced lineages within a species' native range and highlights the need for monitoring intraregional genotype translocations.

## Graphical abstract

In this study, we document a rapid and extensive invasion of the eastern China lineage (haplotype P) of the common reed, Phragmites australis, within its native range on the Yunnan‐Guizhou Plateau. This case is significant as it challenges the conventional paradigm that views invasion threats primarily from exotic species. By integrating field surveys, chloroplast haplotype analysis, and species distribution modeling, we demonstrate how anthropogenic habitat modification (e.g., “returning farmland to wetland”) and the introduction of a nonlocal, competitive genotype via constructed wetlands have jointly driven a large‐scale ecological transformation of a protected lakeshore within a single decade.

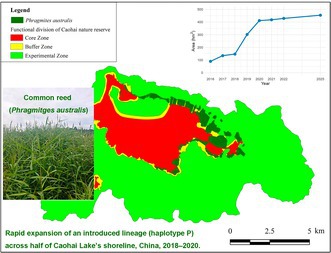

## 1 Introduction

Biological invasions, driven largely by anthropogenic activities, pose a significant threat to biodiversity and ecosystem stability worldwide (Seebens et al. 2025). Among wetland macrophytes, the distinctions between native and invasive species are frequently blurred, driven by close phylogenetic relatedness and shared adaptive traits (Larkin 2024). A particularly understudied phenomenon is that of “native invasion” wherein species become invasive within their own historical ranges following human‐induced environmental change (Carey et al. 2012; Zhao et al. 2024; Paudel et al. 2025). Globally, lakes have experienced rapid ecological changes over the past decade, with satellite remote sensing revealing significant increases in algal blooms and shifts in aquatic vegetation (Hou et al. 2022). The common reed, *Phragmites australis* (Cav.) Trin. ex Steud., may offer an ideal model to study this special invasion process for wetland plants (Meyerson et al. 2025). While widely recognized as a serious invasion in North America due to introduced lineages (Saltonstall 2002; Meyerson and Cronin 2013; Liu et al. 2018), its potential for rapid expansion within its native range, especially in Asia, remains poorly understood.

*Phragmites australis* possesses a suite of traits that predispose it to invasiveness, including clonal integration, allelopathic potential, and broad environmental tolerance (Meyerson et al. 2025). These traits enable the species to form monodominant stands that displace native vegetation and alter habitat structure. Critically, its invasion success is strongly lineage‐dependent (Saltonstall 2002; Lambertini et al. 2012; Meyerson and Cronin 2013). A well‐established chloroplast DNA haplotype system has revealed clear phylogeographic structure across its native and introduced range (Saltonstall 2016). For example, in China, northern China is dominated by haplotypes O and M (the latter being the invasive lineage in North America); eastern China is characterized by the octoploid haplotype P, and southwestern China hosts native haplotypes U and I (An et al. 2012; Tanaka et al. 2017; Liu et al. 2022). This genetic differentiation underscores that invasion outcomes may depend not only on species‐level traits, but also on lineage‐specific adaptations.

While traditionally a natural component of wetland ecosystems in its native range, *P australis*, or specific lineages thereof, is increasingly expanding into novel habitats beyond its historical distribution, largely facilitated by widespread anthropogenic disturbance (Tanaka et al. 2017; Raghuvanshi et al. 2023; Ran et al. 2025; Fouad et al. 2025). Recent advances in lake research emphasize integrated monitoring frameworks that combine remote sensing, modeling, and ground‐based observations to track ecosystem changes and support management decisions (Qiu et al. 2025). This trend is of particular concern in Guizhou Province, a biodiverse karst region in southwestern China characterized by scarce natural lakes and highly fragmented wetlands. Although populations of *P. australis* in Guizhou have historically been sparse and largely confined to constructed wetlands (Tanaka et al. 2017), recent observations indicate noticeable proliferation in the province's largest lake, Caohai Lake, likely linked to anthropogenic habitat modifications (Ran et al. 2025). However, systematic studies are still lacking to quantify its current distribution and genetically trace the origins of these expanding populations.

To address these knowledge gaps, this study investigates the distribution and lineage identity of *P. australis* in the Caohai Lake basin, a critical wetland system in Guizhou. We combine extensive field surveys with chloroplast DNA haplotype analysis to: (1) map the distribution of *P. australis* ; (2) identify the dominant haplotypes present and trace their phylogeographic origins relative to known regional lineages; and (3) discuss the conservation implications of common reed expansion for biodiverse karst wetland ecosystems in southwestern China.

## 2 Materials and Methods

### 2.1 Study Region

This study was conducted at Caohai Lake (26°49′ N, 104°17′ E), the largest natural freshwater lake in Guizhou Province, China, situated on the Yunnan–Guizhou Plateau at 2171 m above sea level. The lake has a karst‐derived, irregular palm‐shaped basin formed in 1857, with a normal water surface area of approximately 31 km^2^, an average depth of 2 m, and a maximum depth of 5 m. The region experiences a northern subtropical monsoon climate, characterized by mean annual precipitation of 950.9 mm and 1805 h of sunshine.

The lake and its surrounding wetlands now form a National Nature Reserve covering 120 km^2^, serving as a critical wintering habitat for 11 first‐class protected bird species, including the Black‐necked Crane ( *Grus nigricollis* ) and Hooded Crane ( *Grus monacha* ). However, the ecosystem has undergone substantial anthropogenic impacts, including drainage and reclamation in the 1970s that drastically shrank the lake area, followed by restoration initiatives since 1982 that recovered the open water to about 25 km^2^. Since 2015, comprehensive measures such as returning farmland to wetland and upgrading wastewater treatment have been implemented (Mu et al. 2024).

### 2.2 Habitat Suitability Estimation

To estimate the habitat suitability habitat for *P. australis* at Caohai, we employed the MaxEnt (Maximum Entropy) model with the occurrence records of *P. australis* in China via the online platform PPDC (an online platform for the prediction of plant distributions in China; https://sdp.iflora.cn/) (Qiu et al. 2024). The model was run using the platform's default parameters and its built‐in database of species occurrences and environmental variables for China. The resulting prediction provides a habitat suitability index ranging from 0 (completely unsuitable) to 1 (optimally suitable).

### 2.3 Field Survey

**FIGURE 1:**
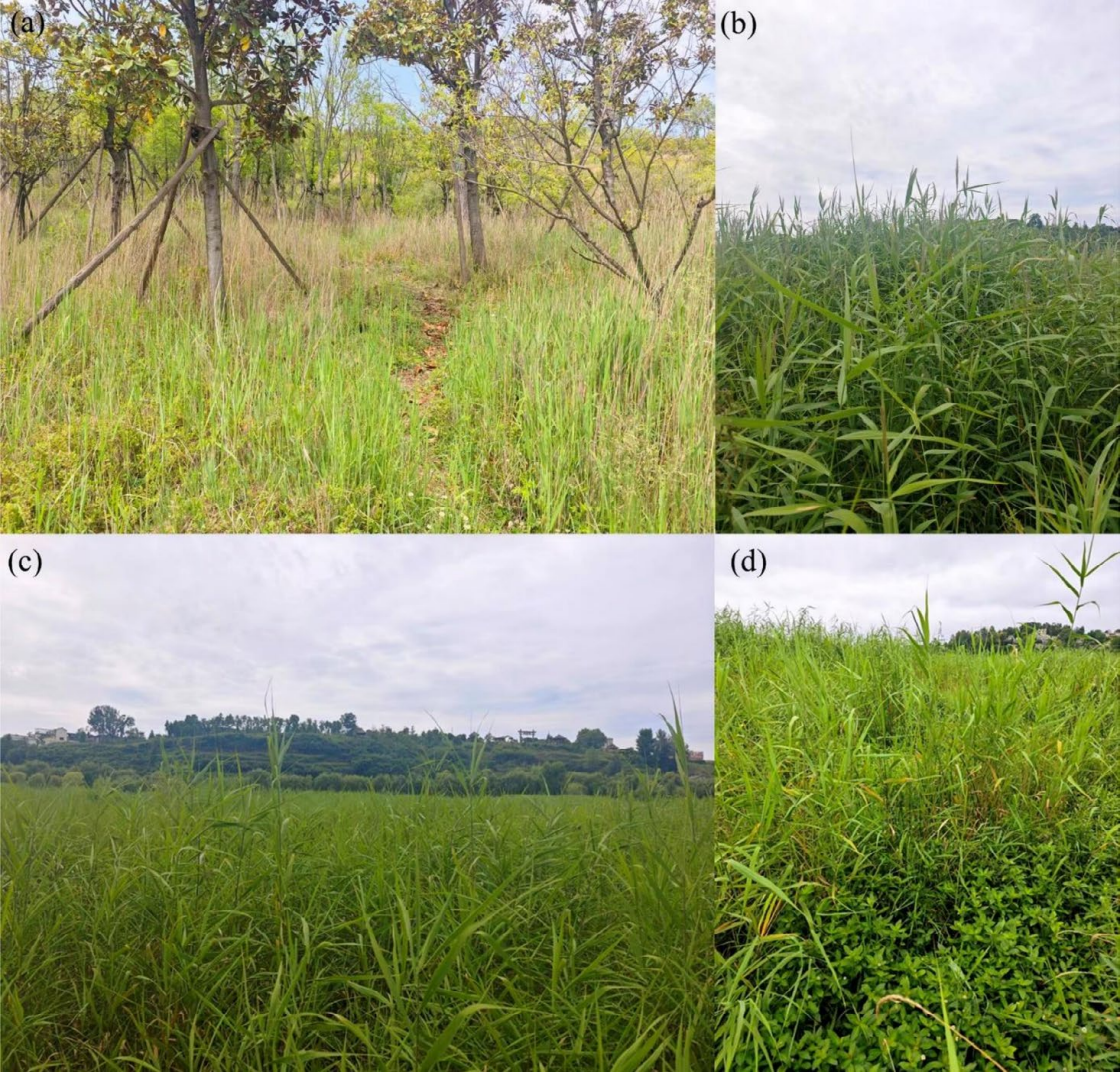
Representative habitats and community types of *Phragmites australis* in the Caohai Lake region. (a) *P. australis* growing in a forest understory on a northern hillside of the lake, one of the earliest colonized areas during its expansion; (b) Close‐up and (c) distant views of a monodominant *P. australis* stand, illustrating its high coverage; (d) A mixed community of *P. australis* and another invasive plant, *Alternanthera philoxeroides* (alligator weed), reflecting interactions among nonnative species in the lakeshore habitat.

A comprehensive field survey of the *P. australis* population within the Caohai National Nature Reserve was conducted throughout the 2025 growing season (Figure 1). To accurately delineate the distribution range of reed patches, we employed an integrated approach that prioritized ground‐based verification. Ground surveys were carried out by research teams who walked transects across the study area, visually confirming the presence of *P. australis* and recording stand boundaries using handheld GPS devices. Observations from elevated vantage points, facilitated using binoculars and spotting scopes, allowed for comprehensive visual coverage of extensive and inaccessible wetland areas. High‐resolution satellite imagery (accessed via Google Earth) was used as a base map to assist with navigation during fieldwork and to provide a spatial reference for manually digitizing the GPS‐confirmed stand boundaries. It is important to note that remote sensing was not used for automated species classification or vegetation index calculation, as current technology cannot reliably distinguish *P. australis* from other cooccurring herbaceous species in this mixed‐community wetland. The final distribution map was therefore generated by integrating ground‐verified GPS data with manual digitization against the satellite base map. The historical range of *P. australis* (2015–2022) was extracted from a previous paper in Chinese (Ran et al. 2025).

### 2.4 Haplotype Analysis

**FIGURE 2:**
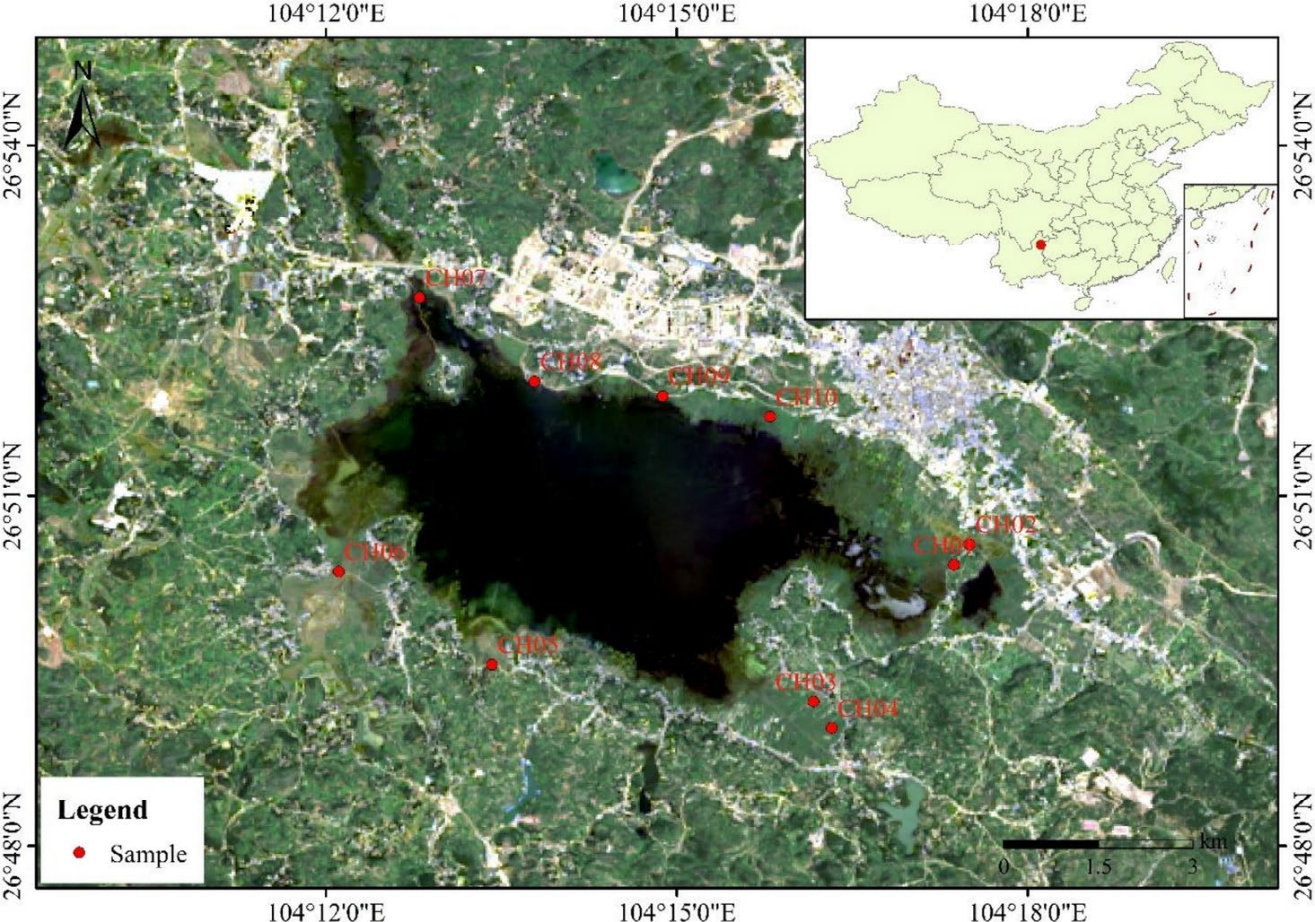
Sampling locations for chloroplast haplotype analysis of *Phragmites australis* in Caohai Lake. The map shows the geographical positions of the 10 sampling sites distributed across the lake basin. Base map data were sourced from the Geospatial Data Cloud, utilizing Landsat 8 satellite remote sensing imagery (data identifier: LC08_L2SP_129041_20190624_20200827_02_T2; path: 129, row: 41). Chloroplast DNA sequencing of the *trnT*‐*trnL* and *rbcL*‐*psaI* regions confirmed that all 52 sampled individuals belonged to haplotype P, indicating a genetically homogeneous invasion of the eastern China lineage throughout the study area.

A total of 52 leaf samples of *P. australis* were collected during the 2025 growing season. To maximize the capture of genetic variation and ensure representation of diverse habitat conditions, our sampling strategy encompassed 10 distinct sites (Table 1) strategically distributed across the three functional zones (core, buffer, and experimental areas) of Caohai National Nature Reserve (Figure 2). These sites covered the major habitat types present in the lake basin, including shallow shorelines in the experimental area, swampy wetlands in the buffer zone, and open‐water littoral zones in the core area, where sampling was conducted via artificial boardwalks to ensure accessibility. At each site, 4–7 individual plants were sampled, with each individual separated by at least 5 m to minimize the risk of sampling the same clonal individual multiple times. Fresh leaf tissue from each sample was silica‐dried in the field.

**TABLE 1:** Sampling site information and sample sizes for *Phragmites australis* haplotype analysis in Caohai Lake.

Population	Latitude (°N)	Longitude (°E)	Sample size
CH01	26.840122	104.289511	4
CH02	26.843059	104.291768	5
CH03	26.820621	104.269502	7
CH04	26.816841	104.272086	5
CH05	26.825894	104.22366	6
CH06	26.839167	104.201841	4
CH07	26.878288	104.21335	4
CH08	26.866306	104.229728	5
CH09	26.864209	104.247996	5
CH10	26.861255	104.263305	7

Fresh leaf tissue from each sample was silica‐dried in the field. Approximately 30 mg of dried leaf tissue from each sample was placed in a 2.0‐mL tube with a 5‐mm glass bead, flash‐frozen in liquid nitrogen, and ground to a fine powder using a Tissuelyser II (Qiagen, Germany) at 30 Hz for 1 min. Genomic DNA was subsequently isolated from the homogenized powder using the DNAsecure Plant Kit (Tiangen, China), following the manufacturer's protocol. The integrity of the extracted DNA was visually assessed on 1% agarose gels, while the purity and concentration were measured using a NanoDrop 2000 spectrophotometer (Thermo Scientific, USA).

Chloroplast haplotype identification was performed based on the established nomenclature for *P. australis* (Saltonstall 2002, 2016). Two noncoding chloroplast DNA (cpDNA) regions, trnT‐trnL and rbcL‐psaI, were amplified via polymerase chain reaction (PCR) using published primers and cycling conditions (Liu et al. 2022). The resulting PCR products were purified and sequenced bidirectionally. The sequences were assembled, aligned, and compared with reference haplotypes in the GenBank database to assign a specific haplotype to each individual.

## 3 Results

**FIGURE 3:**
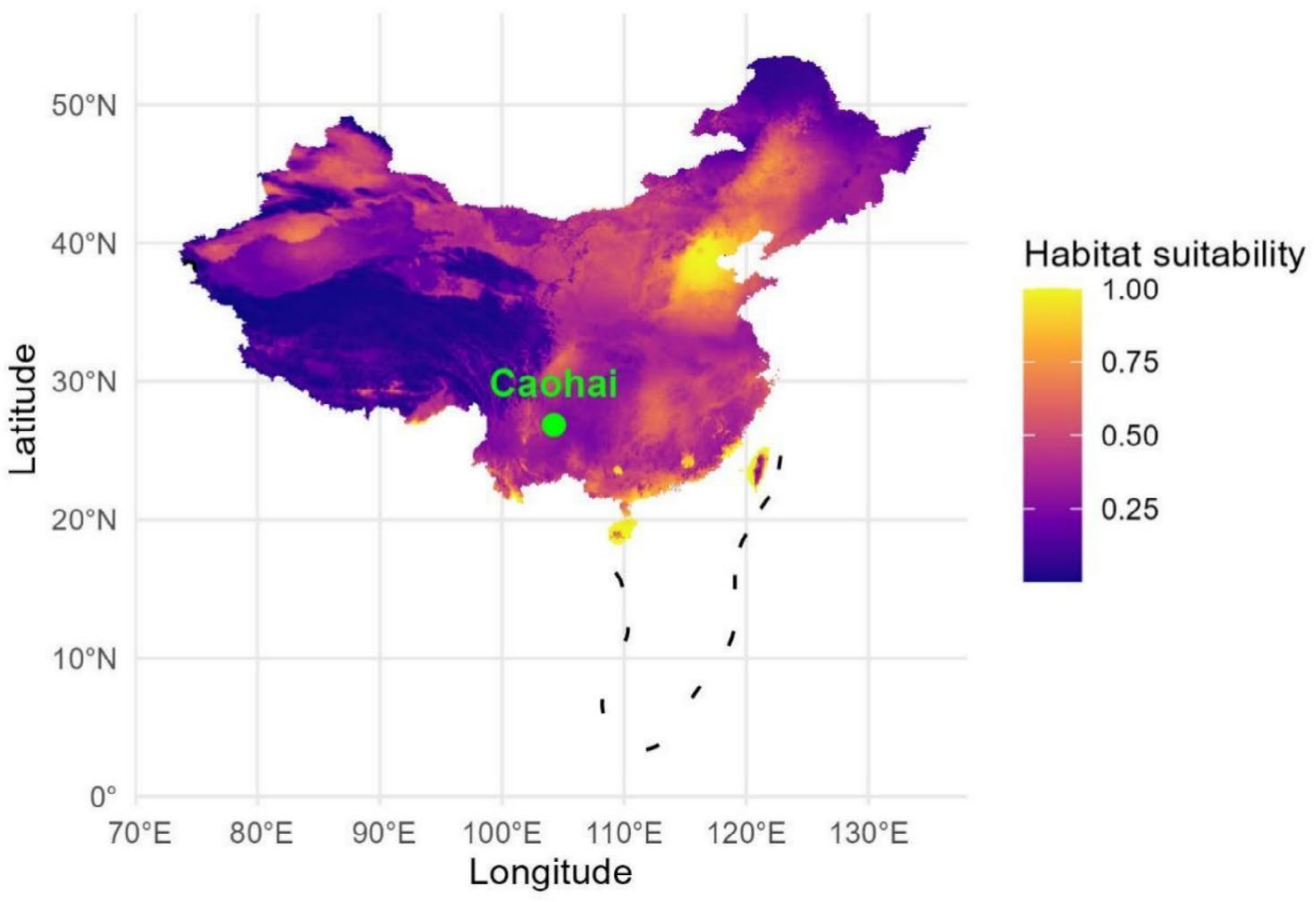
Location of Caohai Lake and the habitat suitability of *Phragmites australis* in China based on species distribution model (MaxEnt) via the online platform PPDC. Map lines delineate study areas and do not necessarily depict accepted national boundaries.

The distribution model indicated that the environmental conditions of Caohai Lake are marginally suitable for *P. australis* (Figure 3). The specific habitat suitability index for the Caohai Lake area was 0.354. This result can be accessed in detail under the task ID 153D03DB‐22AA‐424B‐A88E‐F73F856B7A31 on the PPDC platform.

**FIGURE 4:**
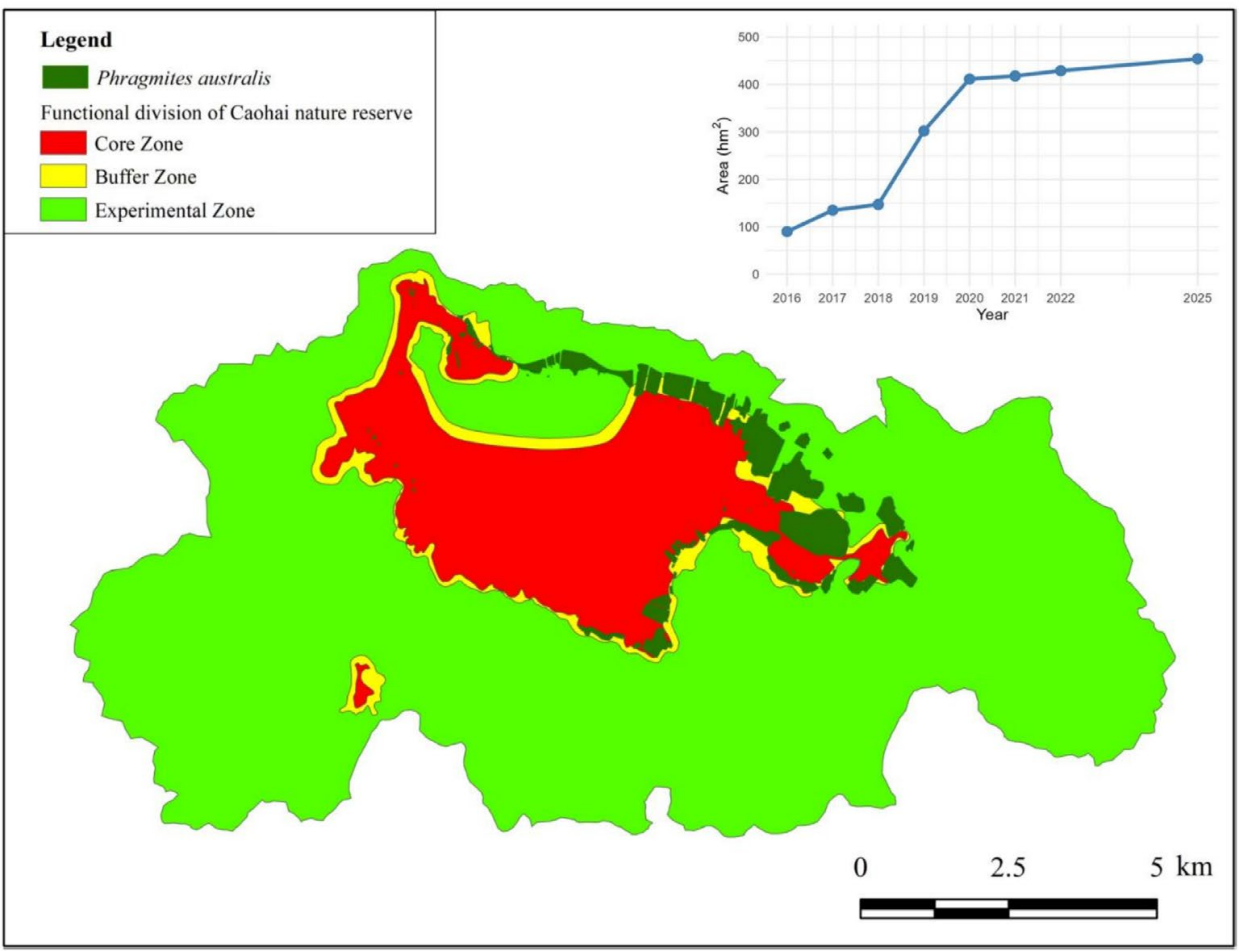
Spatial distribution and temporal dynamics of *Phragmites australis* in Caohai National Nature Reserve. The main map illustrates the distribution of reed patches across the core, buffer, and experimental zones of the reserve in 2025, as derived from field surveys and remote sensing interpretation. The inset line chart in the upper right depicts the temporal change in total reed area since 2016, highlighting a pronounced expansion phase between 2018 and 2020, followed by recent stabilization.

The integrated field and remote sensing survey conducted in 2025 determined the total distribution area of *P. australis* in the Caohai Lake basin to be 454.09 ha, accounting for 3.8% of the total area of the National Nature Reserve. Analysis of the spatial distribution across the different functional zones of the reserve revealed that the reed patches were unevenly distributed: 11.30% were in the core zone, 25.43% in the buffer zone, and the majority, 63.28%, were found in the experimental zone (Figure 4). A comparison with historical data from Ran et al. (2025) covering 2015–2022 indicated that the most significant period of reed expansion occurred between 2018 and 2020. Since this period of rapid growth, the overall distribution area has stabilized, with no substantial changes observed in recent years.

Chloroplast haplotype analysis was conducted on all 52 collected *P. australis* individuals using two noncoding regions, *rbcL‐psaI* and *trnT‐trnL*. The *rbcL‐psaI* region was successfully sequenced for all samples and was identical (haplotype R5) across all individuals. However, for the *trnT‐trnL* region, 11 out of 52 samples yielded sequencing data of insufficient quality due to repetitive sequences that frequently cause difficulties in Sanger sequencing. For the remaining 41 samples where both markers were successfully sequenced, the *trnT‐trnL* sequences were also identical (haplotype T1) and matched the combined haplotype P. Given that all samples shared R5 sequences matching haplotype P, and that all successfully sequenced *trnT‐trnL* fragments confirmed this assignment, we conclude that all 52 individuals belong to haplotype P. This haplotype represents the introduced, invasive eastern China lineage, a genetically distinct octoploid lineage.

## 4 Discussion

Our study documents the rapid transformation of Caohai Lake's littoral zone by *P. australis* haplotype P, an octoploid lineage native to eastern China that has expanded to cover approximately half of the lake's shoreline within a single decade (Figure 4). This extensive colonization contrasts sharply with ecological niche model predictions of low habitat suitability, underscoring how acute anthropogenic disturbances, such as altered hydrology, nutrient enrichment, and shoreline modification, can create transient invasion windows that override conventional climatic suitability thresholds. As a classic R‐strategist, haplotype P proved preadapted to exploit these newly disturbed habitats, with its expansion accelerating markedly between 2018 and 2020, coinciding with regional environmental changes (i.e., “returning farmland to wetland”) (Mu et al. 2024).

The invasion was enabled not only by habitat disturbance but also by the lineage's distinct genomic background. As an octoploid, haplotype P likely possesses enhanced competitive ability compared to other haplotypes (Liu et al. 2022; Yin et al. 2024; Guo et al. 2025; Haldan et al. 2025). This polyploidy‐derived trait suite appears to have preadapted the lineage to conditions in the modified Caohai littoral zone, facilitating its dominance despite the presence of locally adapted flora. The invasion success of *P. australis* haplotype P can be attributed to its higher nutrient acquisition efficiency and growth advantage, as evidenced by its significantly lower C:N and N:P ratios compared to native species (Bai et al. 2025).

Critically, the occurrence of haplotype P in this region is unequivocally linked to human‐mediated introduction. Given its geographical disjunction from eastern China (Liu et al. 2022), natural dispersal is highly improbable. Instead, the widespread use of this lineage in constructed wetlands for wastewater treatment and restoration across Guizhou Province provided the most plausible introduction pathway. These engineered systems, often stocked with nonlocal genotypes for their utility in nutrient uptake, have inadvertently served as invasion epicenters (Lambert et al. 2016; Tanaka et al. 2017), dispersing rhizomes or seeds into natural waterbodies like Caohai Lake. Besides, human‐induced water‐level fluctuations have facilitated the invasion of *P. australis* by eliminating submerged vegetation (Chao et al. 2024), thereby reducing competition and creating vacant ecological niches.

We acknowledge that this study did not include direct measurements of environmental variables or long‐term growth dynamics, which represents an important limitation. While we have documented the rapid expansion of *P. australis* haplotype P in Caohai Lake, the specific environmental drivers facilitating this expansion remain to be empirically determined. Previous studies have documented significant anthropogenic modifications to the lake's hydrology and water quality (Chao et al. 2024; Mu et al. 2024), but whether these factors differentially favor haplotype P over native vegetation requires further investigation. Additionally, long‐term fixed‐location monitoring of annual‐scale growth dynamics, including plant height, coverage, and biomass, is needed to fully characterize the expansion process and its interannual variability. Future research should integrate long‐term monitoring of key environmental parameters to elucidate the causal relationships between environmental change and lineage‐specific expansion.

Beyond environmental drivers, several mechanistic questions regarding dispersal and population genetics warrant future investigation. First, the specific pathways of propagule dispersal for haplotype P remain to be elucidated. Field observations suggest that water flow (northwest to southeast) and prevailing northerly and northeasterly winds during autumn and winter may facilitate rhizome fragment and seed dispersal, respectively, consistent with observed expansion patterns from northern shores to southwestern and northwestern areas (Ran et al. 2025). However, quantitative simulations integrating hydrodynamic and wind field data are required to test these hypotheses. Second, the use of only chloroplast markers limits our ability to assess nuclear genetic diversity, mating systems, and clonal structure. Given that haplotype P is octoploid and may exhibit high genomic compatibility (Liu et al. 2022), future studies employing nuclear markers such as SSRs or SNPs will be essential to determine whether the population is dominated by asexual reproduction and to refine the invasion source. Finally, spatial heterogeneity in expansion dynamics, particularly the correlation between expansion rates in different functional zones and human disturbance intensity (e.g., proximity to “returning farmland to wetland” engineering sites or sewage discharge points), remains unexplored. Spatial overlay analysis along these lines would provide critical insights into the anthropogenic drivers of this invasion.

Despite these unanswered questions, our findings have immediate conservation implications. This case highlights the underappreciated risk of “native invasions” (Carey et al. 2012) wherein intraspecific lineages, introduced outside their historical range, become aggressive colonizers following environmental change. It underscores the necessity of recognizing intraspecific genetic identity in invasion risk assessment (Saltonstall 2002; Meyerson et al. 2025) and calls for stricter oversight of plant sourcing in ecological engineering (Tanaka et al. 2017; Liu et al. 2022). By reporting this rapid expansion event now, we aim to raise awareness among researchers and policymakers while acknowledging that finer‐scale mechanistic questions require dedicated future studies. The use of nonlocal genotypes, even within a species' broad native range, requires careful scrutiny to prevent similar scenarios in other vulnerable ecosystems undergoing anthropogenic transformation.

## 5 Conclusions

The rapid colonization of approximately half of Caohai Lake's shoreline by the eastern China lineage (haplotype P) of *P. australis* exemplifies a “native invasion” driven by human activity. This expansion reflects a convergence of anthropogenic disturbances, including wetland restoration and the introduction of nonlocal genotypes via constructed wetlands, and the lineage's preadapted traits, likely derived from its polyploid origin. Our findings challenge the conventional focus on exotic species in invasion biology and emphasize the need to regulate the use of nonlocal genotypes in ecological restoration, even within a species' native range. The case of Caohai Lake serves as a critical warning for other ecosystems undergoing comparable human‐modified changes.

## Author Contributions

**Wangjun Li:** conceptualization (equal), formal analysis (equal), investigation (equal), methodology (equal), writing – original draft (equal). **Lele Lin:** data curation (equal), formal analysis (equal), methodology (equal). **Yuhui Wang:** data curation (equal), investigation (equal), methodology (equal). **Wenlong Sun:** visualization (equal), writing – review and editing (equal). **Weihua Guo:** conceptualization (equal), data curation (equal), methodology (equal). **Xiaolong Bai:** investigation (equal), methodology (equal), writing – review and editing (equal). **Lele Liu:** formal analysis (equal), investigation (equal), methodology (equal), software (equal), writing – review and editing (equal).

## Funding

This work was supported by the National Natural Science Foundation of China (No. 32470388), the Shandong Provincial Natural Science Foundation (ZR2024QC197), the Bijie Science and Technology Project (bikelianhe[2023]10), and the Project of Guizhou Science and Technology Fund (qiankehejichu‐ZK‐[2024]key077). Lele Liu is supported by the Cyrus Tang Foundation through the Tang Scholar Program.

## Conflicts of Interest

The authors declare no conflicts of interest.

## Data Availability

The code and data supporting the findings of this study are publicly available the Zenodo Digital Repository: https://doi.org/10.5281/zenodo.18408838.
